# Ketamine as an Adjunct Therapy in Acute Severe Asthma: An In-Depth Review of Efficacy and Clinical Implications

**DOI:** 10.7759/cureus.62483

**Published:** 2024-06-16

**Authors:** Jacob Epperson, Zoraize Moeez Athar, Mahnoor Arshad, Edward Y Chen

**Affiliations:** 1 Internal Medicine, BronxCare Health System, New York, USA

**Keywords:** asthma exacerbations, refractory asthma, status asthmaticus, efficacy of ketamine, pediatric asthma, poor asthma outcomes

## Abstract

Acute severe asthma, formerly named status asthmaticus, is defined as a life-threatening asthma exacerbation that is refractory to the current standards of treatment such as the use of beta-agonists and epinephrine. This complication of asthma affects up to 15% of individuals with asthma and despite critical care treatment and hospitalization, there remains a staggeringly high 10-18% mortality rate in an intensive care unit setting. The addition of ketamine to the arsenal of acute severe asthma treatment due to its rapid onset, variable routes of administration, and overall improved clinical efficacy in treatment-refractory cases has been well investigated and documented. Ketamine's anti-inflammatory properties, bronchodilatory effects, and well-documented history contribute to its ability to provide a significant clinical asthma score (CAS) reduction and improvement on pulmonary readings, such as peak expiratory flow (PEF), while providing a well-researched adverse effect profile. This article serves to analyze and review the benefits and risks of incorporating ketamine into the standard treatment regimen for patients suffering from acute severe asthma and discusses the implications of such implementation.

## Introduction and background

Asthma is a chronic inflammatory airway disease that involves recurrent wheezing, dyspnea, chest pain, and coughing. Current treatment guidelines for acute asthma attacks with mild to moderate symptoms include beta-agonist therapy and steroids [1]. Acute severe asthma, formerly known as status asthmaticus, is defined as severe asthma unresponsive to repeated courses of beta-agonist therapy or subcutaneous epinephrine [2].

Acute severe asthma, further defined by the British Guidelines for Asthma and the Global Initiative for Asthma (GINA), is an asthma exacerbation that presents with any of the following factors: peak expiratory flow (PEF) 50% best or predicted, respiratory rate ≥ 30/minute, heart rate ≥ 120 beats/minute, and inability to complete full sentences in a single breath [2]. The American Thoracic Society and European Respiratory Society define a severe asthma exacerbation as the necessity of urgent action in order to prevent a serious outcome such as hospitalization or death [3]. They expand on this further by stating it should include at least one of the following: systemic corticosteroid use, an increase from baseline maintenance dose for at least three days, or a hospital or emergency department visit due to asthma requiring systemic corticosteroids [3,4].

Per the Centers for Disease Control and Prevention (CDC) in 2021, the overall prevalence of asthmatic individuals in the United States decreased from 7.9% in 2017 to 7.7% [1]. Of those affected by asthma, multiple observational studies have shown a more dramatic increase in incidence in African American women over the past 17 years [1]. Of those who suffer from status asthmaticus, research shows that there is a higher prevalence of those in a lower socioeconomic status and individuals who live alone [1,5]. Of those who require hospitalization for asthma, roughly 5-15% of individuals will require care in an intensive care unit (ICU) with status asthmaticus. Of those patients, there is a reported mortality rate of 10-18% [1,2]. Globally, there are roughly 350 million individuals who suffer from asthma, with a proposed mortality rate of less than 0.5% [4]. Of the global deaths, there is a similar correlation between socioeconomic status and mortality rates, which has been documented by the Global Asthma Report [4].

Histopathological changes of respiratory epithelial cells show inflammation and cellular infiltration of eosinophils (and some neutrophils), activated CD4+ T lymphocytes, and mast cells. Intraluminal mucus plugs, also known as Curschmann’s spirals, composed of mucin glycoproteins, plasma proteins, epithelial cells, and cellular debris are characteristically found [6].

The underlying pathophysiology of asthma is multifactorial, including genetic, environmental, and infectious causes, which lead to chronic inflammation in the lower airways. This inflammation causes the closure of the lower airways during exhalation. There is a notable decrease in the amount of air that can be forcibly exhaled in one second (FEV1) and PEF ≤ 50%, whereas the residual capacity may increase as much as 400% of the normal expected value [1,4]. An increased functional residual capacity and characteristic air trapping exacerbates a ventilation-perfusion (V/Q) mismatch along with hypoxia and arterial hypoxemia, thus creating an anaerobic environment resulting in lactic acidosis [1,4,7]. In severe asthma cases and status asthmaticus, hypercapnia and concurrent respiratory muscle fatigue lead to worsening alveolar hypoventilation [4].

Current guidelines set by GINA for severe or difficult-to-treat asthma in 2023 state that in addition to high-dose inhaled corticosteroids, long-acting beta-2 agonists, add-on treatment with long-acting antimuscarinic antagonists [8]. Leukotriene receptor antagonists, low-dose azithromycin, and targeted biologic therapy should be initiated. In acute severe asthma exacerbations leading to emergency department visitation, GINA recommends short-acting beta-agonists, ipratropium bromide, intravenous (IV) magnesium, high-dose inhaled corticosteroids, and oral or IV corticosteroids. Intramuscular epinephrine is indicated in addition to standard therapy for acute severe asthma in the setting of anaphylaxis or angioedema but is not routinely indicated or beneficial for other exacerbations [8,9]. 

While standard guideline-mediated therapy can be effective for the majority of severe cases, some patients may have worsening respiratory distress requiring invasive ventilation or other rescue agents. The use of anesthetic agents such as isoflurane, sevoflurane, halothane, and ketamine has been found to have bronchodilatory effects [10]. While the risk versus benefit profile of the fluorinated gasses has caused them to fall out of favor, ketamine remains an option for use in severe acute asthma exacerbations.

Methodology

A comprehensive search was conducted in databases, including PubMed and Google Scholar, to identify studies published from 1987 to 2024 that evaluated the effectiveness of ketamine in adults and children with status asthmaticus. The search was restricted to studies published in English, and the most recent search was conducted in January 2024. The keywords used for the search included "ketamine," "status asthmaticus," "asthma," “severe acute asthma,” “ketamine in asthma,” and “ketamine refractory asthma.” The PubMed search yielded 47 articles, and the Google Scholar search yielded an additional 6960 articles.

A manual review of the bibliographies of the retrieved articles was performed based on pre-specified inclusion and exclusion criteria to identify relevant studies. The outcomes of interest were the effects of ketamine on respiratory parameters and vital signs, clinical status, need for invasive ventilation or supplemental oxygen, adverse effects, and mortality.

The inclusion criteria for this review included primary articles, including randomized controlled trials (RCTs), systematic reviews, observational studies, animal studies, and cohort studies that evaluated the role and effectiveness of ketamine as a bronchodilator in status asthmaticus. Reference lists and bibliographies of eligible peer-reviewed articles were also searched for relevant material.

Conversely, case reports, case series, articles published before 1987, non-full-text articles or partial abstracts, and non-English literature were excluded. Studies and publications on ketamine for alternative treatments other than or not related to asthmatic patients were also excluded. After reviewing the full texts of relevant studies, those not fulfilling the review's objective were excluded, leaving 22 articles for literature analysis. Guidelines from applicable health societies were also included.

## Review

First synthesized in 1962 and approved for use in the United States in 1970, ketamine has been a common medication used in anesthesia due to its dissociative effects. While it's not commonly used in the treatment of acute severe asthma, several key characteristics of the medication make it quite suitable for the treatment of severe exacerbations: it has a rapid onset of action with high bioavailability and can be administered intravenously, intramuscularly, and nebulized [11-13]. 

**Figure 1:**
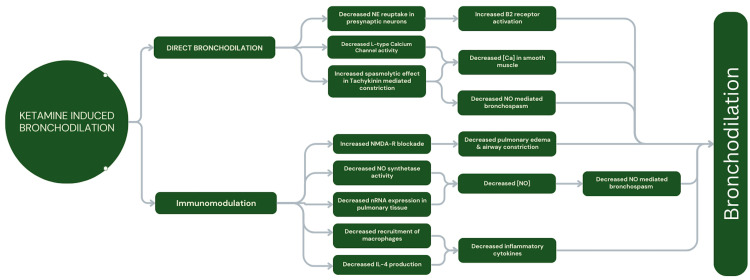
Ketamine's bronchodilatory properties

Ketamine’s high bioavailability and rapid onset of action, reaching a peak plasma concentration within 60 seconds after administration, can be beneficial in asthma in part due to its diverse array of pathophysiological properties (Figure 1). It has been demonstrated to antagonize N-methyl-D-aspartic acid (NMDA) receptors in the lungs and airways, which induces bronchoconstriction [10]. It also reduces nitric oxide levels by down-regulating nitric oxide synthetase in pulmonary tissue. Ketamine has been shown to interfere with the recruitment of macrophages and cytokine production, which are known contributors to asthma exacerbation, thereby reducing interleukin-4 and, thus, bronchoconstriction [10].

In addition to ketamine’s anti-inflammatory properties, it has been shown to directly cause smooth muscle relaxation by three distinct mechanisms: catecholamine-induced smooth muscle bronchodilation, calcium-mediated smooth muscle relaxation, and vagal nerve mediation. Ketamine increases synaptic catecholamine levels by blocking norepinephrine reuptake into presynaptic sympathetic neurons [14]. This acts on beta-2 receptors and leads to smooth muscle-mediated bronchodilation [10]. Anticholinergic effects on bronchial smooth muscle via ketamine use inhibit vagal outflow. This decrease in vagal signaling interrupts L-type calcium channel efflux, resulting in airway smooth muscle relaxation [10,14,15]. Tachykinin, a neural peptide released from vagal C-fibers, increases smooth muscle contraction, epithelial secretion, and epithelial inflammation [16]. Ketamine has been shown to have spasmolytic effects when tachykinin-mediated smooth muscle constriction has occurred [14]. This mechanism is currently under further research, as it is a central component of the inflammatory changes in asthma [10,14], thus making ketamine a suitable abortive medication in status asthmaticus.

Its role as a bronchodilator has been studied well in pediatrics and adults. Binsaeedu et al. conducted studies on pediatric patients with status asthmaticus who were unresponsive to conventional therapy. The patients received a loading dose of 1 mg/kg/hr intravenously for 15 minutes followed by a continuous infusion at 0.75 mg/kg/hr for 1 hour. The results of this demonstrated a significant decrease in mean clinical asthma score (CAS) from 14.2 to 10.5 after 10 minutes of infusion, further reducing to a CAS of 9.5 after 1 hour [11]. Other findings documented were a decrease in respiratory rate and a significant improvement in PEF and blood oxygen saturation [11]. A similar result was found by Youssef et al. when a retrospective analysis examining the effectiveness of ketamine infusions on 17 children who were mechanically ventilated for refractory bronchospasm [17]. In this analysis, the ratio of partial pressure of oxygen in arterial blood (PaO2) to the fraction of inspiratory oxygen concentration (FIO2) revealed a significant increase in PaO_2_/FIO_2_ from 116 ± 55 before ketamine to 174 ± 92, 269 ± 151, and 248 ± 124 at 1, 8, and 24 hours after the infusion (p< 0.0001) [17]. Additionally, measured dynamic compliances increased significantly from 5.78 ± 2.8 cm^3^/cmH_2_O to 7.5 ± 3.39, 7.29 ± 3.37, and 8.58 ± 3.69 after 1, 8, and 24 hours (p<0.0001) [17]. 

In a prospective observational study to evaluate the effect of adding ketamine to standard emergent therapy for status asthmaticus, Petrillo et al. deduced a significant decrease in CAS, respiratory rate, and oxygen requirement, but there was no significant improvement in PEF [10,18]. Another study, performed by Heshmati et al., investigated the use of ketamine in addition to standard abortive therapy in status asthmaticus. By using an arterial blood gas analyzer, PaO_2_ and partial pressure of carbon dioxide in arterial blood (PaCO_2_) were measured, as well as mean peak airway pressure via ventilator analysis. It was found that mean airway pressure and PaCO_2 _decreased significantly (p<0.005) and PaO_2_ increased significantly (p<0.005) after 15 minutes and 2 hours of ketamine infusion [10,19].

Although many studies, such as the ones mentioned above, illustrate a beneficial picture, few randomized controlled trials do not support the use of ketamine over guideline-based therapy. Two different studies, performed by Howton et al. and Allen et al., compared a total of 117 individuals aged 18-65 years randomized to ketamine or placebo with acute asthma exacerbation. In Howton et al.’s analysis, there was a significant reduction in PEF (p=0.004), FEV1 (p=0.001), and respiratory rate (p=0.001), but there was no significant difference between the two groups [10,20], leading to the conclusion that ketamine has no bronchodilatory effect. Allen et al. rated the comparison groups on a 15-point pulmonary index score that failed to show any measurable improvement to standard therapy (0.4; 95% CI -0.4-1.3) [10,20]. A 2022 systematic review done by La Via, investigated the effects of ketamine as an adjunct therapy for severe refractory asthma on oxygenation and respiratory parameters. Their analysis concluded that there was no statistically significant benefit from the administration of ketamine in patients with refractory asthma [21].

The relative safety of ketamine has been long studied and is constantly revisited as continued research progresses. Of the known side effects, a few key reactions were prevalent in each of the studies: hypertension, visual hallucinations, dysphoria, skin flushing, and increased tracheobronchial secretions, potentially leading to aspiration. Hypertension generally resolved without treatment within 10-15 minutes of infusion. Petrillo et al. described the hemodynamic stability of patients while under the use of ketamine and, as noted in previous studies, the hypertension seen during the infusion resolved in a similar timeframe or by the end of the infusion [10,11,18]. A more comprehensive safety analysis and systematic review performed by Migita et al. showed that ketamine therapy was associated with fewer respiratory adverse events, defined as a decreased need for breathing cues and a decreased need for supplemental oxygen compared with hemodynamic stability and the maintenance of spontaneous respirations and airway reflexes at several dosages [13,22]. This was further supported by Green et al. in a safety profile of ketamine in rural hospitals, which showed an adverse effect, defined as death, cardiac arrest, apnea, laryngospasm, or aspiration, a ratio of 19/12,844 or 0.15% (95% CI 0.08-0.24%) [23].

For a comprehensive review of the literature used, refer to Table 1. 

**Table 1:** Summary of literature review on ketamine's use in asthma [11-15,17-21]

Author, Year	Number of patients, age	Population / Study Type	Intervention	Biomarkers of Improvement	Results
Allen et al. 2005	N = 68, Pediatric	Double-blinded, randomized, placebo-controlled trial	IV bolus 0.2 mg/kg followed by a 2-hour infusion at 0.5 mg/kg/hr	Pulmonary index score	No significant difference between groups
Binsaeedu et al. 2023	5 studies on pediatric patients	Systematic review using PubMed and Google Scholar on studies on pediatric patients	IV bolus followed by continuous infusion	Vital parameters, oxygen saturation, dynamic compliance, peak expiratory flow, FIO2, PaO2	Ketamine shows promise as a bronchodilator with improvement in respiratory parameters but further evidence is required
Esmailian et al. 2018	N = 92, Adult	Single-blind, randomized controlled trial	IV bolus with 0.3, 0.4, or 0.5 mg/kg in addition to standard treatment vs standard treatment plus placebo	Peak expiratory flow rate	Ketamine treatment with 0.4 mg/kg led to a significant increase in the peak expiratory flow rate
Farshadfar et al. 2021	N = 35, Adult	Double Blind randomized clinical trial	Nebulized ketamine 0.1-0.3 ml/kg versus magnesium sulfate 2g over 20 min	Peak expiratory flow rate	The mean peak expiratory flow rate was significantly increased in both males and females (p<0.001)
Goyal et al. 2013	20 articles with 244 adults and pediatric patients	Systematic review using MEDLINE, EMBASE, Google Scholar, and Cochrane	IV bolus 0.1 - 2 mg/kg followed by continuous infusion 0.15 - 2.5 mg/kg/hr	Vital parameters, blood gas, oxygen saturation, pressure requirements, dynamic compliance, FIO2	Improved outcomes with the use of ketamine in acute severe asthma unresponsive to standard treatment
Heshmati et al. 2003	N = 11, Adult	Prospective observational	IV bolus 1 mg/kg followed by continuous infusion of 1mg/kg/hr for 2 hours	Peak airway pressure, PaCO2, PaO2	Mean peak airway pressure and PaCO2 decreased significantly and PaO2 significantly increased (p<0.005)
Hirota et al. 1996	5 animal experiments	Animal model testing ketamine and its isomers on the tone of tracheal strips	Ketamine vs effects of adrenaline, CaCl2, and Bay K 8644	Tracheal tone	Ketamine led to the relaxation of contracted airway smooth muscle
La Via et al. 2022	7 total studies, Adult and pediatric	Systematic review on PubMed and EMBASE selecting prospective studies	IV bolus 0.1 - 2.0 mg/kg followed by continuous infusion 0.3 - 2.0 mg/kg/hr	Peak inspiratory pressure, airway resistance, lung compliance, PaO2, PaCO2, peak expiratory flow, FEV1	Does not support the use of ketamine in refractory severe acute asthma
Migita et al. 2006	8 studies with 1086 pediatric patients studied	Systematic review of randomized controlled trials on pediatric emergency department patients	Ketamine + midazolam versus fentanyl + midazolam therapy versus propofol + fentanyl	Safety analysis measuring adverse events, breathing cues, need for supplemental oxygen	- Ketamine + midazolam patients had less pain and anxiety but longer recovery times. - Decreased need for breathing cues, decreased need for supplemental oxygen, decreased desaturation events (p<0.01)
Nedel et al. 2022	N = 45, Adult	Randomized, single-center, evaluator-blinded, parallel-group trial	IV bolus 2 mg/kg followed by continuous infusion 2 mg/kg/hr	Airway resistance, positive end-expiratory pressure, dynamic compliance	Ketamine was not associated with improvement in ventilatory measures associated with bronchospasm
Petrillo et al. 2001	N = 10, Pediatric	Prospective observational	IV bolus 1 mg/kg followed by continuous infusion of 0.75 mg/kg/hr	CAS, peak expiratory flow, vital signs	Significant decrease in asthma score, improved peak expiratory flow, reduced respiratory rate, enhanced oxygen saturation
Youssef et al. 1996	N = 17, Pediatric	Retrospective chart review	IV bolus 2 mg/kg, followed by continuous infusions of 20-60 mcg/kg per minute	PaO2/FIO2 and dynamic compliance	PaO2/FIO2 ratio significantly increased after infusion at 1, 8, and 24 hours. Dynamic compliance improved (p<0.0001)

Limitations

Our review presents several limitations. First, the number of studies included was low, with most of the studies having a small patient sample size. Second, the design of the review of the studies was not homogenous, as we considered both randomized and non-randomized studies and prospective and retrospective studies. Third, we analyzed data from both pediatric and adult patient populations together, thus possibly facing a risk of bias. Finally, some of the studies reviewed were over 20 years old, which could introduce information that is no longer accurate or current.

## Conclusions

The introduction of ketamine into the standards of treatment for acute severe asthma offers a promising outlook on an otherwise high-mortality medical emergency. While many studies have documented its efficacy in rapidly improving the benchmark indicators of acute severe asthma, such as CAS and PEF, some have shown no statistically significant difference between ketamine and standard treatment. Further research is required to fully grasp the mechanisms in which ketamine can benefit individuals afflicted with acute severe asthma as well as to illuminate potential risks and remediations of such.

## References

[1] Chakraborty RK, Basnet S (2024). Status Asthmaticus. https://www.ncbi.nlm.nih.gov/books/NBK526070/.

[2] Afessa B, Morales I, Cury JD (2001). Clinical course and outcome of patients admitted to an ICU for status asthmaticus. Chest.

[3] Reddel HK, Taylor DR, Bateman ED (2009). An official American Thoracic Society/European Respiratory Society statement: asthma control and exacerbations: standardizing endpoints for clinical asthma trials and clinical practice. Am J Respir Crit Care Med.

[4] Kostakou E, Kaniaris E, Filiou E (2019). Acute severe asthma in adolescent and adult patients: current perspectives on assessment and management. J Clin Med.

[5] Rousseau MC, Conus F, El-Zein M, Benedetti A, Parent ME (2023). Ascertaining asthma status in epidemiologic studies: a comparison between administrative health data and self-report. BMC Med Res Methodol.

[6] Papiris SA, Manali ED, Kolilekas L, Triantafillidou C, Tsangaris I (2009). Acute severe asthma: new approaches to assessment and treatment. Drugs.

[7] Shah R, Saltoun CA (2012). Chapter 14: acute severe asthma (status asthmaticus). Allergy and Asthma Proceedings.

[8] (2023). Global Initiative for Asthma. Global strategy for asthma management and prevention, 2023. https://ginasthma.org/wp-content/uploads/2023/07/GINA-2023-Full-report-23_07_06-WMS.pdf.

[9] Gayen S, Dachert S, Lashari BH (2024). Critical care management of severe asthma exacerbations. J Clin Med.

[10] Goyal S, Agrawal A (2013). Ketamine in status asthmaticus: a review. Indian J Crit Care Med.

[11] Binsaeedu AS, Prabakar D, Ashkar M, Joseph C, Alsabri M (2023). Evaluating the safety and efficacy of ketamine as a bronchodilator in pediatric patients with acute asthma exacerbation: a review. Cureus.

[12] Esmailian M, Koushkian Esfahani M, Heydari F (2018). The effect of low-dose ketamine in treating acute asthma attack; a randomized clinical trial. Emerg (Tehran).

[13] Farshadfar K, Sohooli M, Shekouhi R, Taherinya A, Qorbani M, Rezaei-Kojani M (2021). The effects of nebulized ketamine and intravenous magnesium sulfate on corticosteroid resistant asthma exacerbation; a randomized clinical trial. Asthma Res Pract.

[14] Hirota K, Sato T, Rabito SF, Zsigmond EK, Matsuki A (1996). Relaxant effect of ketamine and its isomers on histamine-induced contraction of tracheal smooth muscle. Br J Anaesth.

[15] L'Hommedieu CS, Arens JJ (1987). The use of ketamine for the emergency intubation of patients with status asthmaticus. Ann Em Med.

[16] Steinhoff MS, von Mentzer B, Geppetti P, Pothoulakis C, Bunnett NW (2014). Tachykinins and their receptors: contributions to physiological control and the mechanisms of disease. Physiol Rev.

[17] Youssef-Ahmed MZ, Silver P, Nimkoff L, Sagy M (1996). Continuous infusion of ketamine in mechanically ventilated children with refractory bronchospasm. Intensive Care Med.

[18] Petrillo TM, Fortenberry JD, Linzer JF, Simon HK (2001). Emergency department use of ketamine in pediatric status asthmaticus. J Asthma.

[19] Heshmati F, Zeinali MB, Noroozinia H, Abbacivash R, Mahoori A (2003). Use of ketamine in severe status asthmaticus in intensive care unit. Iran J Allergy Asthma Immunol.

[20] Allen JY, Macias CG (2005). The efficacy of ketamine in pediatric emergency department patients who present with acute severe asthma. Ann Emerg Med.

[21] La Via L, Sanfilippo F, Cuttone G (2022). Use of ketamine in patients with refractory severe asthma exacerbations: systematic review of prospective studies. Eur J Clin Pharmacol.

[22] Migita RT, Klein EJ, Garrison MM (2006). Sedation and analgesia for pediatric fracture reduction in the emergency department. A systematic review. Arch Pediatr Adolesc Med.

[23] Green SM, Clem KJ, Rothrock SG (1996). Ketamine safety profile in the developing world: survey of practitioners. Acad Emerg Med.

